# Differential expression of miRNAs in acute myeloid leukemia quantified by Nextgen sequencing of whole blood samples

**DOI:** 10.1371/journal.pone.0213078

**Published:** 2019-03-20

**Authors:** Aakriti Pandita, Poornima Ramadas, Aarati Poudel, Nibal Saad, Ankit Anand, Alina Basnet, Dongliang Wang, Frank Middleton, Diana M. Gilligan

**Affiliations:** 1 Department of Medicine, Division of Hematology/Oncology, Upstate Medical University, Syracuse, New York, United States of America; 2 Department of Neuroscience and Physiology, Upstate Medical University, Syracuse, New York, United States of America; 3 Department of Public Health and Preventive Medicine, Upstate Medical University, Syracuse, New York, United States of America; European Institute of Oncology, ITALY

## Abstract

New approaches are needed for understanding and treating acute myeloid leukemia (AML). MicroRNAs (miRs) are important regulators of gene expression in all cells and disruption of their normal expression can lead to changes in phenotype of a cell, in particular the emergence of a leukemic clone. We collected peripheral blood samples from 10 adult patients with newly diagnosed AML, prior to induction chemotherapy, and 9 controls. Two and a half ml of whole blood was collected in Paxgene RNA tubes. MiRNA was purified using RNeasy mini column (Qiagen). We sequenced approximately 1000 miRs from each of 10 AML patients and 9 controls. In subset analysis, patients with NPM1 and FLT3 mutations showed the greatest number of miRNAs (63) with expression levels that differed from control with adjusted p-value of 0.05 or less. Some of these miRs have been described previously in association with leukemia, but many are new. Our approach of global sequencing of miRs as opposed to microarray analysis removes the bias regarding which miRs to assay and has demonstrated discovery of new associations of miRs with AML. Another strength of our approach is that sequencing miRs is specific for the 5p or 3p strand of the gene, greatly narrowing the proposed target genes to study further. Our study provides new information about the molecular changes that lead to evolution of the leukemic clone and offers new possibilities for monitoring relapse and developing new treatment strategies.

## Introduction

One area of research that has grown explosively in the past 15 years is the study of non-coding RNA. MiRNAs are 19–22 nucleotide long non-coding RNAs which regulate the expression of genes by sequence-specific binding to mRNA to either promote or block its translation [1]. This is a powerful level of epigenetic control for gene expression that can influence the phenotype of a cell [2]. Several authors have examined the role of miRNA in the transformation of hematopoietic stem cells into leukemic cells [3–8]. It is now well established that miRNAs play a role in blocking differentiation of leukemic cells and promoting their unchecked cell division [9,10].

To date, researchers have analyzed miRNA expression in leukemic cells using arrays or RT-PCR. Our work is the first quantitative sequencing of miRNAs found in peripheral blood from patients with newly diagnosed acute myeloid leukemia (AML). We demonstrate here proof of principle for this relatively simple method. We compare the expression levels to normal controls to find statistically significant increase or decrease in levels of expression of approximately 1000 miRNAs. Subset analysis of AML patients with FLT3 or NPM1 mutations yields further information regarding statistically significant changes in expression of specific miRNAs.

## Materials and methods

This protocol was reviewed and approved by the Upstate Medical University Institutional Review Board. Blood was obtained following written informed consent from patients at University Hospital with newly diagnosed AML, prior to start of induction chemotherapy. Blood was also obtained following written informed consent from healthy volunteers involved in patient care at Upstate. 2.5 ml of whole blood was collected into Paxgene RNA preparation tubes and stored at -80C for batch processing. miRNA was purified using a Qiagen miRNA purification kit. The yield and quality of the RNA samples was assessed using the Agilent Bioanalyzer prior to library construction using the Illumina TruSeq Small RNA Sample Prep protocol (Illumina; San Diego, California). Multiplexed samples of RNA that exceed quality control metrics (RIN > 6.0) were run on an Illumina NextSeq500 instrument at a targeted depth of 10 million reads per sample. After filtering and trimming of index and adapter sequences, whole genome alignment of the miR FASTQ reads was performed using the Homo sapiens/hg21 reference genome in the SHRiMPS aligner included in the miRNAs analysis application available in BaseSpace (Illumina), as well as the sRNA Toolbox application suite.

### Statistical method

The analysis of the RNA-seq data was performed following the pipeline available from the *limma* packages [11] in the Bioconductor project [12]. Log2counts per million (logCPM) transformation was applied before normalization and linear model fitting. Empirical Bayes moderation was carried out to obtain robust estimates of gene-wise variability and the final p-values from the linear model with appropriately designed contrasts were adjusted by the Benjamin–Hochberg procedure for a targeted false discover rate of 0.05. Volcano plots and boxplots were used to graphically examine the differences between groups.

## Results

We sequenced all miRNAs in peripheral whole blood from ten patients with newly diagnosed AML and nine normal controls. Table 1 shows the characteristics of the ten patients who entered the study. There were five males and five females, ages ranged from 42 to 87. Initial white blood cell (WBC) count ranged from 1.1 x 10^3^/ul to 88 x 10^3^/ul, with blast percentage of 3.8 to 73. Phenotype was determined by standard hematopathology staining for surface markers. For all ten patients, we recorded presence (pos) or absence (neg) of NPM1 or FLT3 mutations and cytogenetic analysis.

**Table 1:** Characteristics of patients in study.

Age	Gender	Initial WBC x 1000/ul	Blast %	Phenotype	NPM1	FLT3	Cytogenetics
55	M	1.5	73	Acute biphenotypic T/myeloid	neg	pos	normal
42	F	9	17	Erythroleukemia from CML	neg	neg	complex
55	F	29	13	Acute monocytic leukemia	pos	pos	normal
87	F	88	18	Acute myelomonocytic leukemia	pos	pos	normal
57	M	10.9	35	Acute myeloid leukemia	pos	pos	trisomy 8
72	M	28	4.5	Acute myeloid leukemia	pos	neg	normal
59	M	1.8	4	Acute erythroid leukemia	neg	neg	normal
72	M	30	23	Acute myeloid leukemia	neg	neg	normal
87	F	2.3	42	Acute myeloid leukemia	neg	neg	normal
49	F	1.1	3.8	Acute promyelocytic leukemia	neg	neg	15:17

We obtained sequence for 996 miRNAs and averaged the amount of each miRNA for the ten AML patients and the nine controls. Levels of expression were compared with a t-test and the adjusted P-value was calculated for each miRNA. In our first analysis of all ten patients versus controls, we identified only thirteen miRNAs with expression levels that showed a statistically significant difference (adjusted P-value less than 0.05) [13].

**Fig 1:**
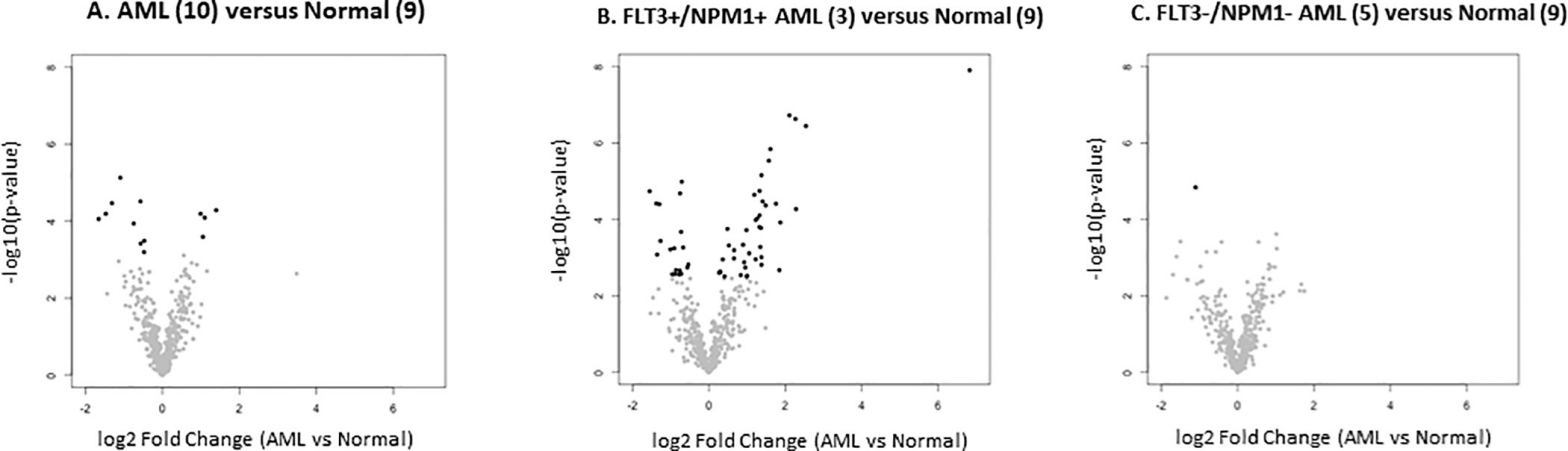
Volcano plots showing differences in miRNAs expressed in leukemia versus control peripheral blood. X-axis: log2 Fold Change Y-axis: -log10(p-value). A. AML versus Normal (n = 10) B. FLT3+/NPM1+ AML versus Normal (n = 3) C. FLT3-/NPM1- AML versus Normal (n = 5).

We then performed subset analysis of our data and found that patients who were double negative for mutations (NPM1- and FLT3-) had only one miRNA with adjusted P-value less than 0.05, while patients who were double positive for mutations (NPM1+ and FLT3+) had 63 miRNAs with adjusted P-value less than 0.05 [14]. Results are shown in Fig 1 (volcano plots).

In Fig 1, the volcano plot for the comparison of all ten AML patients versus control is shown in panel A. Log2 Fold Change is shown on the x-axis and–log10 adjusted p-value is shown on the y-axis. Points in dark font indicate miRNAs with statistically significant log fold change and adjusted p-value (four are increased in AML, nine are decreased in AML). Panel B shows the volcano plot for the subset of patients who were double positive (NPM1+/FLT3+, n = 3), with 63 miRNAs having statistically significant change in expression. Similarly, panel C shows the volcano plot for the subset of AML patients who were double negative (NPM1- /FLT3-, n = 5) with only one statistically significant change in miRNA expression. Clearly, the subset analysis identified a greater number of miRNAs with statistically significant change in expression in the AML patients with NPM1 and FLT3 mutations (double positive).

Table 2 shows the first twenty (out of 996) miRNAs that were identified, listed by rank order of adjusted P-value, lowest to highest, in comparison of AML versus control samples. The center list is from all ten AML patients versus nine controls. The first 13 show statistically significant difference with adjusted p-value less than 0.05. The list on the left is from the comparison of double negative patients (NPM1-/FLT3-) (n = 5) to nine controls. Only hsa-miR-328-3p shows a statistically significant difference with adjusted p-value less than 0.05. Interestingly, some of the other miRNAs in the top twenty do show a statistically significant change in the double positive patients (identified by color bars). The list on the right is from the comparison of double positive patients (NPM1+/FLT3+) (n = 3) to nine controls. There are 63 (out of 996) miRNAs with statistically significant difference in expression and adjusted p-value less than 0.05, but only the top twenty (lowest adjusted p-values) are shown here. The color bars are used to demonstrate which miRNAs are found in two out of the three, or all three samples sets.

**Table 2:** Differential expression of miRNAs in patients versus controls ranked by adjusted P-value.

NPM1- FLT3- (n = 5)	adj.P.Val	AML (N = 10)	adj.P.Val	NPM1+ FLT3+ (n = 3)	adj.P.Val
hsa-miR-328-3p	0.014	hsa-miR-328-3p	0.007	hsa-miR-10a-5p	1.24E-05
hsa-miR-24-3p	0.078	hsa-miR-106b-3p	0.011	hsa-miR-146b-5p	7.79E-05
hsa-let-7i-5p	0.078	hsa-let-7i-5p	0.011	hsa-miR-181a-3p	7.79E-05
hsa-miR-34a-5p	0.078	hsa-miR-181a-3p	0.011	hsa-miR-155-5p	8.95E-05
hsa-miR-1229-3p	0.078	hsa-miR-409-3p	0.011	hsa-miR-199b-5p	0.0002
hsa-miR-181a-3p	0.089	hsa-miR-10b-5p	0.011	hsa-miR-24-3p	0.0004
hsa-miR-3200-5p	0.089	hsa-miR-24-3p	0.011	hsa-miR-19b-3p	0.0009
hsa-miR-106b-3p	0.089	hsa-miR-126-5p	0.011	hsa-miR-425-5p	0.0012
hsa-miR-15a-5p	0.104	hsa-miR-3200-5p	0.013	hsa-let-7a-3p	0.0018
hsa-miR-744-5p	0.135	hsa-miR-23a-3p	0.026	hsa-miR-4301	0.0018
hsa-let-7d-5p	0.135	hsa-miR-323b-3p	0.029	hsa-miR-181a-2-3p	0.0018
hsa-miR-23a-3p	0.135	hsa-miR-652-3p	0.032	hsa-miR-335-5p	0.0018
hsa-miR-625-5p	0.135	hsa-miR-181a-2-3p	0.049	hsa-miR-10b-5p	0.0024
hsa-miR-10b-5p	0.142	hsa-miR-22-3p	0.056	hsa-miR-328-3p	0.0024
hsa-miR-126-5p	0.186	hsa-miR-3960	0.074	hsa-miR-142-3p	0.0024
hsa-miR-15b-3p	0.193	hsa-miR-625-5p	0.077	hsa-miR-3615	0.0024
hsa-miR-125b-2-3p	0.193	hsa-miR-181b-5p	0.08	hsa-miR-23a-3p	0.0025
hsa-miR-409-3p	0.193	hsa-miR-4772-3p	0.087	hsa-miR-27a-3p	0.0029
hsa-miR-3960	0.193	hsa-miR-744-5p	0.087	hsa-miR-181b-5p	0.0041
has-miR-4301	0.193	has-miR-146b-5p	0.087	has-miR-125b-2-3p	0.0047

**Fig 2:**
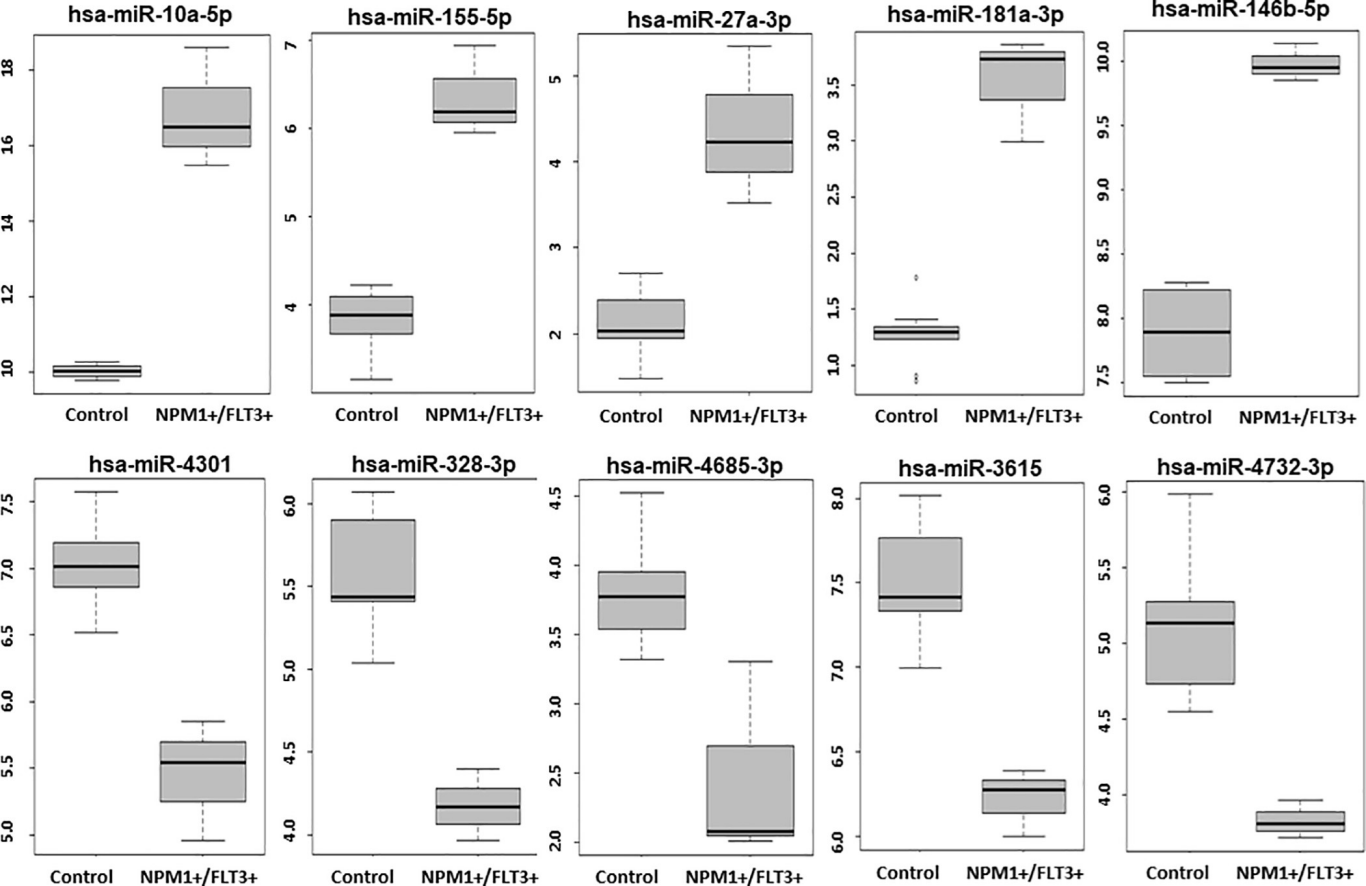
Boxplots showing relative expressions levels of ten miRNAs with statistically significant difference between control and AML NPM1+/FLT3+ patient samples.

Fig 2 shows boxplots of the relative expression levels for the ten miRNAs showing greatest increase or greatest decrease when comparing NPM1+/FLT3+ AML to controls. The boxplots show the average expression level (log2 counts per million) for each of ten miRNAs in controls versus NPM1+/FLT3+AML blood samples.

Table 3 shows all 63 miRNAs that had statistically significant differences in expression between NPM1+/FLT3+ patients and controls, listed in decreasing order of log2Fold Change (log2FC). Positive values of log2FC indicate increased expression in the AML patients, while negative values of log2FC indicate decreased expression in the AML patients. MiRs that have not previously been associated with AML are indicated with asterisks.

**Table 3:** Differential expression of miRNAs between NPM1+/FLT3+ AML patients and controls.

miRNA	Log2FC	miRNA	Log2FC
hsa-miR-10a-5p	6.830569383	hsa-miR-339-5p	0.654592192
hsa-miR-155-5p	2.540153625	hsa-miR-378c	0.652454498
hsa-miR-27a-3p	2.282394076	hsa-miR-371b-5p	0.521138094
hsa-miR-181a-3p	2.26616154	hsa-miR-500a-3p *	0.481850139
hsa-miR-146b-5p	2.107437782	hsa-miR-378d	0.407412804
hsa-miR-21-3p	1.870878887	hsa-miR-181b-3p	0.360659064
hsa-miR-340-5p	1.844418662	hsa-miR-1910-5p *	0.295654388
hsa-miR-142-3p	1.755215629	hsa-miR-195-5p	0.287016156
hsa-miR-199b-5p	1.608767963	hsa-miR-6882-5p *	0.286345176
hsa-miR-24-3p	1.567277012	hsa-miR-3667-5p *	0.285098138
hsa-miR-23a-3p	1.484152009	hsa-miR-3922-3p *	0.279184469
hsa-miR-10b-5p	1.406513049	hsa-miR-548al *	0.268147495
hsa-miR-6503-3p *	1.374880947	hsa-miR-199a-5p	-0.542007567
hsa-miR-19b-3p	1.373298073	hsa-miR-361-3p	-0.564971933
hsa-miR-28-5p *	1.370999597	hsa-miR-106b-3p	-0.677684789
hsa-miR-10a-3p	1.359816952	hsa-miR-425-5p *	-0.711429318
hsa-miR-582-3p *	1.343371672	hsa-miR-532-3p *	-0.726695535
hsa-let-7a-3p	1.325948775	hsa-miR-92a-3p	-0.730022499
hsa-miR-181b-5p	1.325353217	hsa-miR-181a-2-3p	-0.758535453
hsa-miR-30e-3p	1.322709817	hsa-miR-6511b-3p *	-0.768444175
hsa-miR-125b-2-3p	1.258254645	hsa-miR-574-3p *	-0.782318226
hsa-miR-29b-3p	1.224950228	hsa-miR-3605-3p *	-0.866437008
hsa-miR-29a-3p	1.223591909	hsa-miR-3940-3p *	-0.886685286
hsa-miR-335-5p	1.184936831	hsa-miR-3200-5p*	-0.911067568
hsa-miR-3688-3p *	1.054040212	hsa-miR-484 *	-0.961659564
hsa-miR-222-3p	0.996724055	hsa-miR-486-5p	-1.010732451
hsa-miR-769-5p *	0.986880376	hsa-miR-4732-3p *	-1.270122308
hsa-miR-451a	0.981368744	hsa-miR-3615 *	-1.299778948
hsa-miR-1307-5p *	0.94986408	hsa-miR-4685-3p *	-1.35782737
hsa-miR-338-3p	0.918728355	hsa-miR-328-3p	-1.380320316
hsa-miR-223-3p	0.891956558	hsa-miR-4301 *	-1.554755355
hsa-miR-23a-5p	0.830519732		

*indicates miRNAs not previously associated with leukemia

## Discussion

Rather than analyze by microarray which would identify only 300–400 miRNAs, we chose to sequence all miRNAs that were obtained in whole blood samples from patients with newly diagnosed AML and controls. We quantified expression of 996 miRNAs from each patient and control and performed statistical analyses to determine which miRNAs were increased or decreased in AML patients versus normal controls. Subset analysis revealed the most differences in patients with double positive AML (NPM1+/FLT3+ mutations). NPM1 mutations are the most common genetic abnormalities in AML (50–60% of cytogenetically normal AML and 30% of all AML) [15]. Up to one-third of NPM1+ patients also have a mutation in FLT3, which counteracts the favorable prognosis of the NPM1 mutation [16]. The NPM1 gene encodes a 32-kDA protein that is involved in numerous cellular processes. It can function as an oncogene and a tumor suppressor gene depending on expression levels, interacting proteins, and cellular compartmentalization. Dozens of mutations have been described in NPM1 and how they contribute to leukemogenesis is not known. Finding 63 distinct miRNAs either upregulated or downregulated in NPM1+ /FLT3+ AML suggests that there may be multiple molecular pathways disrupted that contribute to the leukemic phenotype.

On our list of miRNAs associated with NPM1+/FLT3+ AML, miR-10a-5p showed the most statistically significant adjusted p-value as well as the highest fold change. This miRNA has been described in patients with NPM1 mutations and high expression levels are associated with good response to induction chemotherapy [17]. More recent studies demonstrate a role for miR-10a/b in regulating the proliferation and differentiation of HL-60 leukemic cells *in vitro* [18].

One miR family that we identified with statistically significant change was the miR-181 family (181a-3p, 181a-2-3p and 181b-5p). This family has been shown by others to be consistently increased in AML patients [8,19,20]. There are numerous possible target mRNAs for the miR-181 family and it has been proposed as a therapeutic target.

Another report has shown that miR-199b is consistently decreased in AML and we also found that to be the case in our patients [21]. We found increased expression of miR-223-3p and this has been shown to regulate granulopoiesis in mice [22].

We compared our list of miRNA differences determined by Nextgen sequencing to lists of miRNA differences found by hybridization arrays or RT-PCR [10, 23–27]. Many of the miRNAs identified by these methods were the same as ours, confirming our ability to generate meaningful data with our approach. Differences in miRNAs identified can be attributed to differences in technique of sample processing (whole blood versus isolated cells or serum). Identification of novel miRNAs in association with AML are likely due to the greater sensitivity of our approach, i.e. sequencing all miRNAs obtained in whole blood.

New therapeutic approaches are being created to target specific miRNAs in leukemic patients [28,29]. As we continue to investigate the role of miRNAs in leukemogenesis, the approaches of diagnosis, treatment, and post-treatment monitoring will be greatly improved.

## Supporting information

S1 Table Differential expression of miRNAs in patients with NPM1+/FLT3+ AML versus control. This table shows the statistical analysis for each of 996 miRNAs for patients with NPM1+/FLT3+ AML versus normal controls. Results are listed in decreasing order of adjusted P. Value.
(XLSX)
